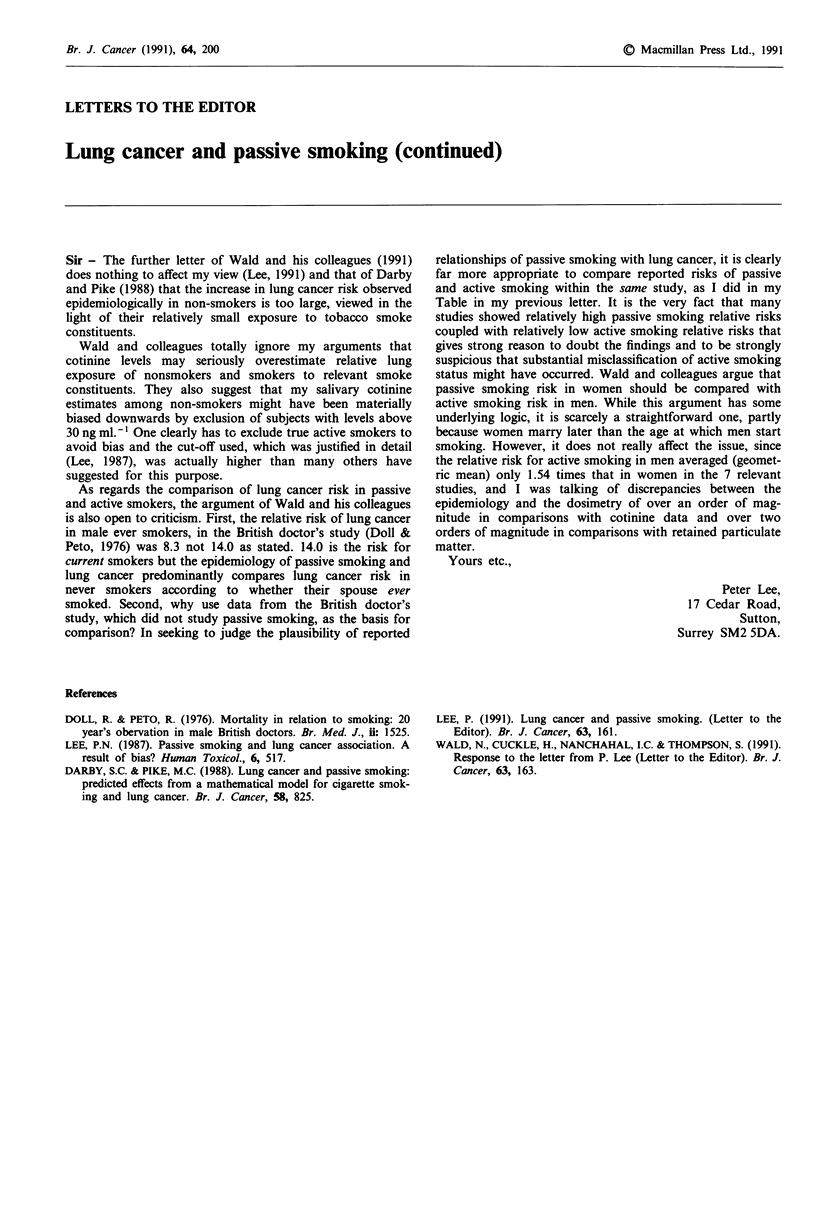# Lung cancer and passive smoking (continued)

**DOI:** 10.1038/bjc.1991.271

**Published:** 1991-07

**Authors:** P. Lee


 © Macmillan Press Ltd., 1991

### LETTERS TO THE EDITOR

### Lung cancer and passive smoking (continued)

**Sir** – The further letter of Wald and his colleagues (1991) does nothing to affect my view (Lee, 1991) and that of Darby and Pike (1988) that the increase in lung cancer risk observed epidemiologically in non-smokers is too large, viewed in the light of their relatively small exposure to tobacco smoke constituents.

Wald and colleagues totally ignore my arguments that cotinine levels may seriously overestimate relative lung exposure of nonsmokers and smokers to relevant smoke constituents. They also suggest that my salivary cotinine estimates among non-smokers might have been materially biased downwards by exclusion of subjects with levels above 30 ng $ml.^{-1}$ One clearly has to exclude true active smokers to avoid bias and the cut-off used, which was justified in detail (Lee, 1987), was actually higher than many others have suggested for this purpose.

As regards the comparison of lung cancer risk in passive and active smokers, the argument of Wald and his colleagues is also open to criticism. First, the relative risk of lung cancer in male ever smokers, in the British doctor's study (Doll & Peto, 1976) was 8.3 not 14.0 as stated. 14.0 is the risk for *current* smokers but the epidemiology of passive smoking and lung cancer predominantly compares lung cancer risk in never smokers according to whether their spouse *ever* smoked. Second, why use data from the British doctor's study, which did not study passive smoking, as the basis for comparison? In seeking to judge the plausibility of reported relationships of passive smoking with lung cancer, it is clearly far more appropriate to compare reported risks of passive and active smoking within the *same* study, as I did in my Table in my previous letter. It is the very fact that many studies showed relatively high passive smoking relative risks coupled with relatively low active smoking relative risks that gives strong reason to doubt the findings and to be strongly suspicious that substantial misclassification of active smoking status might have occurred. Wald and colleagues argue that passive smoking risk in women should be compared with active smoking risk in men. While this argument has some underlying logic, it is scarcely a straightforward one, partly because women marry later than the age at which men start smoking. However, it does not really affect the issue, since the relative risk for active smoking in men averaged (geometric mean) only 1.54 times that in women in the 7 relevant studies, and I was talking of discrepancies between the epidemiology and the dosimetry of over an order of magnitude in comparisons with cotinine data and over two orders of magnitude in comparisons with retained particulate matter.

Yours etc.,

Peter Lee,
17 Cedar Road,
Sutton,
Surrey SM2 5DA.

**References**

DOLL, R. & PETO, R. (1976). Mortality in relation to smoking: 20 year's obervation in male British doctors. *Br. Med. J.*, ii: 1525.

LEE, P.N. (1987). Passive smoking and lung cancer association. A result of bias? *Human Toxicol.*, 6, 517.

DARBY, S.C. & PIKE, M.C. (1988). Lung cancer and passive smoking: predicted effects from a mathematical model for cigarette smoking and lung cancer. *Br. J. Cancer*, 58, 825.

LEE, P. (1991). Lung cancer and passive smoking. (Letter to the Editor). *Br. J. Cancer*, 63, 161.

WALD, N., CUCKLE, H., NANCHAHAL, I.C. & THOMPSON, S. (1991). Response to the letter from P. Lee (Letter to the Editor). *Br. J. Cancer*, 63, 163.